# Comparison of a time-varying covariate model and a joint model of time-to-event outcomes in the presence of measurement error and interval censoring: application to kidney transplantation

**DOI:** 10.1186/s12874-019-0773-1

**Published:** 2019-06-26

**Authors:** Kristen R. Campbell, Elizabeth Juarez-Colunga, Gary K. Grunwald, James Cooper, Scott Davis, Jane Gralla

**Affiliations:** 1 Department of Pediatrics, University of Colorado Anschutz Medical Campus, Aurora, 80045 Colorado USA; 2 Department of Biostatistics and Informatics, University of Colorado Anschutz Medical Campus, Aurora, 80045 Colorado USA; 3 Adult and Child Consortium for Health Outcomes and Delivery Science, University of Colorado Anschutz Medical Campus, Aurora, 80045 Colorado USA; 4 Division of Renal Diseases and Hypertension, University of Colorado Anschutz Medical Campus, Aurora, 80045 Colorado USA

**Keywords:** Bayesian analysis, Interval censoring, Measurement error, Shared random effects

## Abstract

**Background:**

Tacrolimus (TAC) is an immunosuppressant drug given to kidney transplant recipients post-transplant to prevent antibody formation and kidney rejection. The optimal therapeutic dose for TAC is poorly defined and therapy requires frequent monitoring of drug trough levels. Analyzing the association between TAC levels over time and the development of potentially harmful de novo donor specific antibodies (dnDSA) is complex because TAC levels are subject to measurement error and dnDSA is assessed at discrete times, so it is an interval censored time-to-event outcome.

**Methods:**

Using data from the University of Colorado Transplant Center, we investigated the association between TAC and dnDSA using a shared random effects (intercept and slope) model with longitudinal and interval censored survival sub-models (JM) and compared it with the more traditional interval censored survival model with a time-varying covariate (TVC). We carried out simulations to compare bias, level and power for the association parameter in the TVC and JM under varying conditions of measurement error and interval censoring. In addition, using Markov Chain Monte Carlo (MCMC) methods allowed us to calculate clinically relevant quantities along with credible intervals (CrI).

**Results:**

The shared random effects model was a better fit and showed both the average TAC and the slope of TAC were associated with risk of dnDSA. The simulation studies demonstrated that, in the presence of heavy interval censoring and high measurement error, the TVC survival model underestimates the association between the survival and longitudinal measurement and has inflated type I error and considerably less power to detect associations.

**Conclusions:**

To avoid underestimating associations, shared random effects models should be used in analyses of data with interval censoring and measurement error.

**Electronic supplementary material:**

The online version of this article (10.1186/s12874-019-0773-1) contains supplementary material, which is available to authorized users.

## Background

There are nearly 100,000 patients on the U.S. kidney transplant waiting list, with 13 people dying every day while awaiting this life-saving therapy [1]. This national shortage of organs has made it imperative to ensure the longest possible kidney allograft survival for the approximately 20,000 patients that receive a transplant each year, yet over half of kidney grafts fail by 10 years after transplantation [1]. There has been an evolving recognition that antibody-mediated rejection represents one of the prominent barriers to improving long-term graft outcomes [2]. The antibodies that mediate this rejection process, de novo donor-specific antibodies (dnDSA), have been established as an early biomarker for post-transplant adverse kidney events and patients are now screened for dnDSA at regular intervals at most centers around the country [3, 4]. Immunosuppression therapy is likely the most directly modifiable factor in preventing dnDSA. The majority of centers in the U.S. (93%) use Tacrolimus (TAC) as the backbone of their immunosuppression protocols [5], but the drug has a narrow and poorly defined therapeutic range that varies by patient.

The relationship between TAC and dnDSA is still largely unexplored due to numerous complexities. TAC trough levels, or the lowest concentration of drug in the blood prior to the next dose of medication, are measured frequently post-transplant. The measurement error is high because of biological variability in how the drug is absorbed. In addition, TAC is considered an internal covariate in relation to dnDSA since both processes are occurring in the same individual and there may exist a feedback loop in which low TAC levels lead to dnDSA but the suspected presence of dnDSA causes a TAC dose increase. To add to the complexities, screening for dnDSA is conducted only periodically, e.g. every six months to one year, which makes dnDSA an interval censored outcome. It is known that dnDSA have developed during the interval, but the exact timing of dnDSA is unknown. In previous studies, one-dimensional functions of observed TAC values were found to be associated with development of dnDSA; these functions included the coefficient of variation of TAC, the percentage of TAC levels below 5 ng/ml [6], the mean TAC troughs of less than 8 ng/ml and the percentage of time TAC was in therapeutic range [7]. These studies did not address the longitudinal aspect of TAC, the measurement error or possible feedback from dnDSA to TAC, or the interval-censored nature of dnDSA.

We compare two methods to model a heavily interval censored (dnDSA) and a highly variable longitudinal outcome (TAC): (i) a joint model (JM) and (ii) a survival model with a time varying covariate (TVC). It has been shown that when exact event times are known, the presence of measurement error causes the association parameter estimated by a TVC model to be biased towards null [8, 9]. We sought to further this exploration by studying how the amount of measurement error and the width of the censoring intervals around the time-to-event outcome affect the association estimate in JM and TVC. Interval censored time to event outcomes occur frequently in clinical situations, in particular for conditions that are subclinical and detectable only through a specialized assessment. However, to our knowledge, a thorough comparison of TVC versus JM in the presence of differing amounts of measurement error and interval censoring has yet to be performed.

First, we fit a joint model to analyze the association between longitudinal TAC levels and interval censored dnDSA. The literature of joint models of longitudinal and survival outcomes is extensive including textbooks [10] and [11] and comprehensive review articles [12–15]. Although methods have been developed for single survival outcomes [16–19], much less attention has been given to interval-censored outcomes in joint models [20–22]. Gueorguieva et al. developed a joint model for longitudinal and interval censored competing risk dropout outcomes using a family of parametric baseline hazards including Weibull and log-logistic and used maximum likelihood estimation [22]. We build our joint model for TAC and dnDSA based on this work, but with a single time to event interval censored outcome, a correlated error structure within the longitudinal sub-model, and estimation using Markov Chain Monte Carlo (MCMC).

Second, we fit an interval censored survival model with a time-varying covariate (TVC), following the work by Sparling et al. [23]. We compare the estimates of association between TAC and dnDSA, with the hypothesis that the association parameter in the TVC model will be underestimated. Finally, we perform a series of simulation studies to further explore how the two models perform in terms of bias, level and power based on (1) varying strengths of association, (2) varying amounts of measurement error in TAC, and (3) varying degrees of interval censoring in dnDSA.

In the first section, we introduce the kidney transplant study data. Next, the models are formulated and applied to the motivating kidney transplantation dataset. A simulation study comparing the models under different levels of interval censoring and different amounts of measurement error in the longitudinal process is presented and a discussion on the findings and comparison of the models follows.

### Kidney Transplant Study

This retrospective study included patients who were at least 18 years old at time of kidney transplant between September 2007 and December 2013 at the University of Colorado Hospital. Patients were excluded if they had primary non-function, simultaneous liver and kidney transplant, islet cell transplant, had pretransplant DSA, failed to undergo dnDSA screening, or had dnDSA within the first week post-transplant. Informed consent was provided by all patients, and this study was conducted in accordance with the Declaration of Helsinki and was approved by the ethics committee at the University of Colorado (COMIRB 13-3137). No organs or tissues were obtained from prisoners and all kidney transplant recipients received organs in accordance with the United Network of Organ Sharing, the Organ Procurement and Transplantation Network and a local organ procurement organization, the Donor Alliance, as directed by the United States National Organ Transplant Act.

**Fig. 1:**
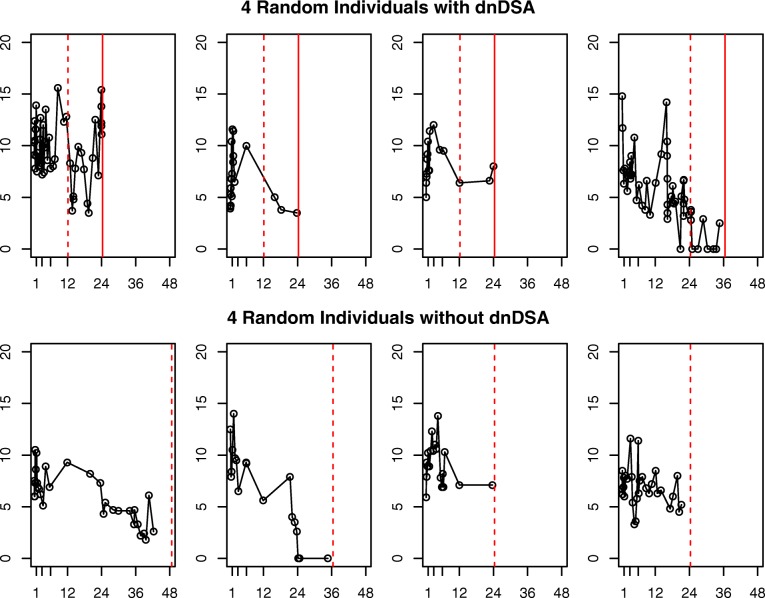
Observed trajectories of four patients who developed dnDSA and four who did not. The x-axis represents time post-transplant, measured in months. The y-axis represents observed TAC level. The black circles represent measured TAC levels, which are connected with a solid black line for visibility. For those who developed dnDSA (1st row), the dotted red line represents the left censoring time and the solid red line represents the time at which dnDSA was detected. For those who did not develop dnDSA (2nd row), the red dotted line represents the last time at which dnDSA was checked and was negative, which is the right censoring time

Five hundred and thirty eight patients met the final inclusion criteria for this analysis. Baseline characteristics of interest are summarized in Table 1. It is hypothesized that age, race, and the degree of Human Leucocyte Antigen (HLA) mismatch between the donor and recipient are associated with development of dnDSA [7]. Post-transplant, each patient was closely monitored and data on TAC trough levels and formation of dnDSA were collected for up to 7 years. Each individual had up to 90 measures of TAC that ranged in value from 0 to 30. No obvious violation of normality assumption in the distribution of TAC was observed (histogram in Additional file 1: Figure S1). Due to the high variability and unreliability of TAC levels within the first week, only TAC levels after day 7 were included in the analysis. TAC levels of zero indicated that a patient was non-compliant and did not take their prescribed TAC dose or a patient was purposefully taken off the drug due to some other medical complication, such as an infection. The number and frequency of TAC measures varied by patient, with an overall median of 22 measurements per person (IQR: 15-32) (Table 1). dnDSA screening was performed at 1, 6, 12 months, annually, and when clinically indicated. Of the 538 patients, 181 developed dnDSA during the study period. For the purpose of this project, dnDSA was treated as a time to event variable, with the event being the first time an individual tested positive for dnDSA. Figure 1 shows the TAC trajectories and time to dnDSA or censoring of 4 random individuals who developed dnDSA and 4 random individuals who did not develop dnDSA.

**Table 1:** Kidney Transplant Study Descriptive Statistics

Summary Measure	No dnDSA (n=357)	dnDSA (n=181)	Overall (n=538)
Male	225 (63%)	111 (61%)	336 (62%)
Age			
*Young*	24 (7%)	23 (13%)	47 (9%)
(<30 years)			
*Middle*	120 (34%)	80 (44%)	200 (37%)
*(30-49 years)*			
*Old*(≥*50 years*)	213 (60%)	78 (43%)	291 (54%)
Ethnicity			
*Caucasian*	265 (74%)	107 (59%)	372 (69%)
*African American*	25 (7%)	26 (14%)	51 (9%)
*Hispanic*	53 (15%)	42 (23%)	95 (18%)
*Other*	14 (4%)	6 (3%)	20 (4%)
Number of HLA Mismatches *median (IQR)*	4 (2, 5)	4 (3, 5)	4 (3, 5)
Total TAC Measurements *median (IQR)*	26 (18, 37)	17 (11, 25)	22 (15, 32)
Months of Followup *median (IQR)*	48 (20, 60)	12 (6, 24)	36 (12, 60)

## Methods

To analyze the association between TAC and dnDSA, we propose a shared random effects model and compare it with a traditional interval censored survival model treating TAC as a time-varying covariate.

### M1: Shared Random Intercept and Slope Model

Suppose there are *N* individuals each measured at *n*_*i*_ time points, *i*=1,...,*n*_*i*_. Let *y*_*ij*_ be the measurement for individual *i* at time *t*_*ij*_, *j*=1,...,*n*_*i*_. We assume that given the vector of individual-specific random intercept and slope (*a*_0*i*_,*a*_1*i*_)^′^, individual outcome measures *y*_*ij*_ are independent and normally distributed with mean *μ*_*ij*_ and residual variance $\sigma ^{2}_{e}$, where

1 $$ \mu_{ij}=f(t_{ij})+\beta_{1}^{\prime}x_{1i}+a_{0i}+a_{1i}t_{ij}.   $$

The time component *t*_*ij*_ can be modeled with any function *f*. Some examples include a linear trajectory, i.e. *f*(*t*_*ij*_)=*b*_0_+*b*_1_*t*_*ij*_; or b-spline basis functions, i.e. $f(t_{ij})=\sum _{k=1}^{K} \beta ^{\prime }_{0k} \psi _{k}(t_{ij})$, where *ψ*_*k*_(*t*_*ij*_) is the *k*^*th*^ of *K* basis functions with coefficient *β*_0*k*_ and the number of basis functions *K* depends on the degree of the splines (e.g. *g*=3) and number of inner knots (*h*=3; *K*=*g*+*h*+1). The vector *x*_1*i*_ represents baseline covariates with regression coefficients *β*_1_. The parameters *a*_0*i*_, *a*_1*i*_ are the random intercepts and slopes that allow individuals to vary in their baseline TAC level and time trend; they are normally distributed with mean zero and a correlated variance/covariance matrix

2 $$ \left[\begin{array}{c} a_{0i} \\ a_{1i} \\ \end{array}\right]\sim N\left(\begin{array}{cc} \left[\begin{array}{c} 0\\ 0 \\ \end{array}\right], \left[\begin{array}{cc} \sigma_{0}^{2} & \rho\sigma_{0}\sigma_{1} \\ \rho\sigma_{0}\sigma_{1} & \sigma_{1}^{2} \\ \end{array}\right] \end{array}\right).  $$

Suppose the same *N* individuals are measured at discrete and varying timepoints for a time to event outcome. The time to event outcome is interval censored and only known to happen between two discrete time points. Let *t*_*Ri*_ denote the time at which the outcome was detected and *t*_*Li*_ indicate the visit time immediately preceding *t*_*Ri*_. The true time to event, *t*_*i*_, lies somewhere in between *t*_*Li*_ and *t*_*Ri*_. The hazard for individual *i* at any time *t* is given by

3 $$ h_{i}(t)=h_{0}(t)\exp(\beta_{0}+\beta_{2}^{\prime}x_{2i}+\lambda_{0} a_{0i}+\lambda_{1} a_{1i})   $$

where *h*_0_(*t*) is the baseline hazard, *β*_0_ is the fixed intercept and scale parameter, *x*_2*i*_ is the vector of fixed covariates and *β*_2_ is its parameter, (*λ*_0_,*λ*_1_)^′^ are the association parameters, and (*a*_0*i*_,*a*_1*i*_)^′^ are the same random terms as above in the longitudinal sub-model (1). The baseline hazard can a number of flexible distributions, including parametric (typically exponential, Weibull, or gamma), piecewise constant or spline forms. The two sub-models (1) and (3) are connected by the shared random intercepts from the survival portion with both the random intercepts and slopes from the longitudinal portion. The first association parameter, *λ*_0_, corresponds to the risk of event for a difference in average longitudinal measure of one unit. Parameter *λ*_1_ corresponds to the risk of event for a unit increase in the slope of the longitudinal model.

Conditional on random effects, *a*_0*i*_,*a*_1*i*_, the log likelihood can be written for each individual:

4 $$ {\begin{aligned} LL_{i}=&\sum_{j=1}^{n_{i}}\{-\frac{1}{2}ln(2\pi\sigma_{e}^{2}) -\frac{1}{2\sigma_{e}^{2}}(y_{ij}-f(t_{ij})-\beta_{1}^{\prime}x_{1i}-a_{0i}-a_{1i}t_{ij})^{2}\} \\ &-I_{Ri}log\{1-F_{i}(t_{Ri})\}+(1-I_{Ri})log\{F_{i}(t_{Ri})-F_{i}(t_{Li})\} \\ \end{aligned}}  $$

where *I*_*Ri*_ is an indicator variable that is 1 when the individual is right censored and 0 when interval censored, *t*_*Ri*_ is the right censoring time, and *t*_*Li*_ is the left censoring time. *F*_*i*_(*t*) corresponds to the cumulative density function at time *t*. Details on how this likelihood is constructed for the Weibull hazard that was used in the results can be found in the Additional file 1.

### M2 and M3: Special Cases of M1

We also consider special cases of M1 where, instead of sharing both the random intercepts and slopes from the longitudinal model (1) with the random intercepts in the survival model (3), only the random intercepts from the longitudinal model are shared with the random intercepts from the survival model. This model is called M2, and the hazard function of the survival model is the special case when *λ*_1_=0 in Equation (3). Model M3 is the special case when *λ*_0_=0 in Equation (3). This model shares the random slope from the longitudinal model with the random intercept from the survival model.

### M4: Time Varying Covariate Survival Model

We also consider an interval censored survival model with a time-varying covariate for this data. Assume that each individual, *i*=1,...,*N*, has longitudinal measurements at update times *t*_*ij*_, where *j*=1,...,*n*_*i*_. Let *z*_*i*_ denote a vector of baseline covariates for individual *i* and *w*_*ij*_ denote the value of the time-varying covariate for individual *i* at update time *j*. The hazard of event for individual *i* at time *j* can be written as:

5 $$ h_{ij}(t)=h_{0tvc}(t_{ij})\exp(\gamma_{0}+\gamma^{\prime}z_{i}+\eta w_{ij})   $$

where *h*_0*tvc*_ is the baseline hazard function (which can be specified as any flexible distribution, as in M1), *γ*_0_ is the fixed intercept, *γ*^′^ is the coefficient vector for baseline covariates, and *η* is the coefficient for the time-varying covariate, *w*_*ij*_. In this case, *w*_*ij*_ is the same covariate as the longitudinal outcome in M1 (*y*_*ij*_), since we are comparing the performance of M1 and M4 in modeling the same outcome, TAC from our motivating kidney dataset.

The likelihood for this model can be written as in Sparling et al. [23]:

6 $$ \begin{aligned} LL_{i}=\prod_{i=1}^{N}\ -I_{Ri}[1-F_{i}(t_{i}|\boldsymbol{\gamma}_{i},\boldsymbol{w}_{i[(t_{R_{i}})]})] \times \\ (1-I_{Ri})[F_{i}(t_{R_{i}}|\boldsymbol{\gamma}_{i},\boldsymbol{w}_{i[(t_{R_{i}})]})-F_{i}(t_{L_{i}}|\boldsymbol{\gamma}_{i},\boldsymbol{w}_{i[(t_{L_{i}})]})] \end{aligned}   $$

where $\phantom {\dot {i}\!}F_{i}(t_{i}|\boldsymbol {\gamma }_{i},\boldsymbol {w}_{i[(t_{i})]})$ is the cumulative density, conditional on the fixed covariate values *γ*_*i*_ and the relevant time-dependent covariate values *w*_*ij*_ up to the right or interval censoring times. The cumulative density can be iteratively calculated based based on equation (5) for each subject, by using the cumulative hazard and survival functions.

### Bayesian Model Fit

Model estimation and inference is based on the Bayesian framework where the hyper-parameters are assigned weakly informative priors. Priors for the parameters are as follows:

$${\begin{aligned} b_{0},b_{1},\beta_{0},\beta_{1}^{\prime},\beta_{2}^{\prime},\lambda_{0},\lambda_{1},\gamma_{0},\gamma^{\prime},\eta & \stackrel{ind}{\sim} N(0,10000) \\ \sigma_{e} &\sim \text{Uniform}(0,100) \\ \alpha,\alpha_{tvc}&\stackrel{ind}{\sim} \text{Gamma}(100,100)\\ \left[\begin{array}{cc} \sigma_{0}^{2} & \rho\sigma_{0}\sigma_{1} \\ \rho\sigma_{0}\sigma_{1} & \sigma_{1}^{2} \\ \end{array}\!\right]^{-1} & \!\sim Wishart\!\left(\begin{array}{c}\!\left[\begin{array}{cc} 0.00001 & 0 \\ 0 & 0.000001 \\ \end{array}\right],2\!\end{array}\right) \\ \end{aligned}} $$

where *u*∼Normal(*μ*,*σ*^2^) with probability density $1/(\sigma \sqrt {2\pi })\exp {\{-(u-\mu)^{2}/2\sigma ^{2}\}}$; *u*∼Uniform(*a*,*b*) with probability density 1/(*b*−*a*) for *a*<*u*<*b*; *u*∼Gamma(*r*,*λ*) with probability density *λ*^*r*^*u*^*r*−1^exp(−*λu*)/ *Γ*(*r*); and *Ω*∼Wishart(*R*,*k*) with probability density (∣*Ω*∣^(*k*−*p*−1)/2^∣*R*∣^*k*/2^ exp{−*Tr*(*RΩ*/2})/(2^*pk*/2^*Γ*_*p*_(*k*/2)) for *k*≥*p*, where *p*=2, *Tr* is the trace function and *Γ*_*p*_ is the multivariate gamma function.

Markov Chain Monte Carlo (MCMC) simulations were performed to estimate the posterior distribution. A Gibbs sampler was used to construct two Markov chains using JAGS software [24] and the runjags package in R [25]. Convergence of the samples was assessed by trace plot inspection, and Gelman and Geweke tests which test for equality of the means of the first and last part of a Markov chain [26]. After a burn-in period of 20,000 and thinning of 40 of 80,000 sampling iterations, 2000 samples per chain were used for inference.

### Model Selection and Comparison

Model selection was carried out using (a) the Deviance Information Criterion (DIC) [27] and (b) the Watanabe-Akaike information criterion (WAIC) introduced by Watanabe [28]. The DIC is a measure calculated based on the deviance, *D*(***θ***)=−2 log*L*(***y***|***θ***), that penalizes for the number of effective degrees of freedom in the model and is defined as $ DIC=\overline {D(\boldsymbol {\theta })}+\rho _{D}=2\overline {D(\boldsymbol {\theta })}-D(\hat {\boldsymbol {\theta }}) $, where $\bar {D}$ and $\hat {\boldsymbol {\theta }}$ are obtained from MCMC output as the mean of the deviance and the posterior mean, respectively. WAIC approximates leave-one-out cross-validation [28] and works well for hierarchical models where the number of degrees of freedom is not obvious [29]. The best model should have the lowest values of DIC and WAIC; differences of at least 5 points will be considered important. We followed the traditional recommendations by Spiegelhalter and Ntzoufras [26, 27] to use the conditional likelihood rather than the marginal to compare model fits. More discussion on this topic can be found in the work by Kapur [30] and in the supplement (Part G) of Juarez-Colunga [31].

## Results

### Models and Model Selection

Models M1-M4 were fitted to the kidney transplant data. The three joint models (M1, M2, M3) had a longitudinal sub-model with TAC as the outcome, a fixed intercept and slope for time (*f*(*t*_*ij*_)=*b*_0_+*b*_1_*t*_*ij*_)) and a random intercept and/or slope for time (depending on the model) for variations across individuals. Spline terms for time were tested, but DIC and WAIC indicated that a linear trend was sufficient. For the survival sub-models, various baseline hazard functions were tested (exponential, Weibull, gamma, piecewise constant) and the Weibull yielded the best model fit (*h*_0_(*t*)=*αt*^*α*−1^, where *α* is the Weibull shape parameter). The Weibull distribution makes intuitive sense for this data, as the hazard can flexibly be increasing or decreasing over time and the risk of dnDSA is thought to decrease as time from transplant progresses. The parameters from the Weibull model can easily be interpreted as the change in log-relative risk. All survival sub-models had time to dnDSA as the interval censored outcome, random intercept(s) associated with the longitudinal sub-model, and a vector of baseline covariates: age, ethnicity, and number of HLA mismatches. See Table 1 for the description of these covariates. Baseline covariates were tested in both the longitudinal and survival sub-models, but none were significant predictors in the longitudinal sub-model, so they were removed.

The interval censored survival model with a time-varying covariate (M4) had dnDSA as the outcome, TAC as the time-varying covariate, and the same baseline covariates as listed above. After considering other baseline hazard functions for M4, we chose the Weibull due to model fit and to compare to M1, so $\phantom {\dot {i}\!}h_{0tvc}(t)=\alpha _{tvc}t^{\alpha _{tvc}-1}$ where *α*_*tvc*_ is the Weibull shape parameter.

Table 2 displays the results of model comparisons based on the DIC and the WAIC. The survival sub-model fits in M1-M3 can be directly compared with the model fit for M4. The best fitting survival model according to both DIC and WAIC is Model 1, which shares both the random intercept and slope from the longitudinal model with the random intercept from the survival model.

**Table 2:** Model Selection Criteria: DIC, and WAIC as defined in the “Model Selection and Comparison” section

Model	DIC	WAIC
M1: Shared Random Intercept/Slope with Intercept	**68943 (67672+1271)**	**68917 (67608+1309)**
M2: Shared Random Intercepts	69052 (67716+1336)	69029 (67675+1354)
M3: Shared Random Intercept with Slope	69104 (67739+1365)	69057 (67691+1366)
M4: TVC Model	1327	1329

The parentheses contain the (linear sub-model fit + survival sub-model fit). Lowest value indicating best model fit is in bold

### Estimation of Associations

Table 3 presents the results from the final model, M1, and the time-varying covariate model, M4. The longitudinal portion of M1 shows that on average, individuals start at a TAC level of about 7 ng/ml. For every one month post-transplant, the average individual’s TAC levels decrease by 0.05 ng/ml. There is a significant negative correlation between the random intercept and slope, *ρ*=−0.38, indicating that individuals with higher starting TAC have a lower slope. The survival portion of the model has a decreasing hazard over time, parameterized by *α*=0.57. The degree of HLA mismatching is significantly associated with dnDSA; for every one unit increase in mismatching, the risk of dnDSA increases 1.26-fold (95% CrI: 1.13, 1.40). Race is also associated with risk of dnDSA; compared to Caucasian individuals, African Americans and Hispanics have a higher risk of dnDSA (2.09 and 1.60, respectively). Age is also a factor, as middle-aged (30-49 years) and older-aged (50+ years) individuals both have a reduced risk of dnDSA compared to individuals under the age of 30 (Hazard Ratios: 0.57 and 0.30, respectively). These covariate effects are all conditional on the same initial level and slope of TAC, so these results are not explained by different TAC levels across HLA mismatches, race or age groups. The first association parameter, *λ*_0_, explains the risk of dnDSA associated with a difference in overall average level of TAC. For every one ng/ml higher average TAC level, the risk of dnDSA lowers 0.64-fold (95% CrI: 0.52, 0.75). The second association parameter, *λ*_1_, explains the risk of dnDSA associated with a difference in the slope of TAC levels. For every 0.05 ng/ml/month higher TAC slope, the risk of dnDSA lowers 0.43-fold (95% CrI: 0.32, 0.58).

**Table 3:** Results from M1 and M4. M1 is the joint model that shares both the random intercept and slope from the longitudinal sub-model with the random intercept from the survival model. M4 is the interval censored survival model with the longitudinal measurements as a time-varying covariate

	M1: Shared Random Effects				M4: Time Varying Covariate				
			Lower	Upper				Lower	Upper
Parameter	Estimate	SD	95% CrI	95% CrI		Estimate	SD	95% CrI	95% CrI
Linear Sub-Model									
*b*_0_	7.24	0.06	7.12	7.36	-	-	-	-	-
*b*_1_	-0.05	0.005	-0.06	-0.04	-	-	-	-	-
$\sigma ^{2}_{e}$	7.44	0.09	7.26	7.63	-	-	-	-	-
$\sigma ^{2}_{0}$	1.66	0.14	1.40	1.94	-	-	-	-	-
$\sigma ^{2}_{1}$	0.004	0.0006	0.003	0.005	-	-	-	-	-
*ρ*	-0.38	0.07	-0.51	-0.24	-	-	-	-	-
Survival Sub-Model									
*α*	0.57	0.06	0.47	0.69	*α*_*tvc*_	0.46	0.03	0.40	0.53
	Hazard					Hazard			
	Ratio					Ratio			
*β*_0_	0.03	0.01	0.01	0.06	*γ*_0_	0.17	0.06	0.08	0.30
*β*_1_ (HLA Mismatch Number)	1.26	0.07	1.13	1.40	*γ*_1_	1.29	0.06	1.17	1.43
*β*_2_ (African American)	2.09	0.56	1.23	3.39	*γ*_2_	1.79	0.40	1.11	2.70
*β*_3_ (Hispanic)	1.60	0.33	1.05	2.31	*γ*_3_	1.67	0.31	1.14	2.33
*β*_4_ (Other Race)	1.14	0.56	0.34	2.41	*γ*_4_	1.12	0.47	0.40	2.20
*β*_5_ (30-49 years)	0.57	0.15	0.32	0.93	*γ*_5_	0.67	0.17	0.42	1.05
*β*_6_ (≥50 years)	0.30	0.08	0.17	0.49	*γ*_6_	0.41	0.10	0.25	0.65
*λ*_0_	0.64	0.06	0.52	0.75	*η*	0.80	0.03	0.75	0.85
*λ*_1_ (per 0.05 change)	0.43	0.07	0.32	0.58	-	-	-	-	-

For M1, the posterior mean and 95% credible intervals are presented for the linear portion of the model, the survival portion of the model, and the association parameters. The longitudinal portion is comprised of a fixed intercept (*b*_0_), a fixed slope (*b*_1_), and a random error term ($\sim N(0,\sigma ^{2}_{e})$). There is also a random intercept ($a_{0i}\sim N(0,\sigma ^{2}_{0})$) and a random slope for each individual ($a_{1i}\sim N(0,\sigma ^{2}_{1})$), which have a correlation parameter *ρ*. The survival model is comprised of fixed covariates (*β*), the Weibull association parameter *α*, and a random intercept for each individual that is related to the random intercept and slope of the longitudinal sub-model through two association parameters. The first association parameter (*λ*_0_) links the two sub-models through their shared random intercepts. The second association parameter (*λ*_1_) links the two sub-models through the longitudinal random slope and the survival random intercept. M4 contains the Weibull scale parameter, *α*_*tvc*_, baseline covariates with parameter *γ*, and the coefficient for the time-varying covariate, *η*

The interval censored survival model M4 does not have a linear sub-model, but instead uses a time-varying covariate for TAC. The scale parameter of the survival model is *α*=0.46, which also results in a decreasing hazard of dnDSA over time. The effects of baseline covariates, *γ*^′^, are similar in magnitude to *β*^′^ in M1. The hazard ratio for the time-varying TAC is *η*=0.80. Within an individual, following a one unit increase in TAC level, the risk of dnDSA decreases 0.80-fold (95% CrI: 0.75, 0.85). Though this is still a significant finding, the effect size of *η* in M4 is smaller than *λ*_0_ in M1. We explore this further in the “Simulation Study” section.

### Goodness of Fit of Kidney Transplant Study

To assess the adequacy of model fit for M1-M3, we simulated 1000 datasets from the posterior predictive distribution and compared them to the observed data. We performed these posterior predictive assessments separately for the longitudinal and survival sub-models. This is usually done through a discrepancy function represented by a scalar or a vector, often represented graphically [32, 33]. For comparing the observed longitudinal TAC data with the posterior distribution of longitudinal measures on given individuals, we have conditioned on the posterior mean of the random effects at the individual level; e.g. for M1: $\hat {a}_{0i}, \hat {a}_{1i}$ in (1) are considered fixed [34]. We compared 1000 posterior predictive TAC trajectories against the observed trajectory for 3 random individuals using M1, M2 and M3. Due to the large variability in TAC levels within and between individuals, the plots are all nearly identical, which is indicative that no one model fits the longitudinal trajectory better than another model. The plots are shown in Additional file 1: Figure S2.

For the survival sub-model predictive check, we created artificial intervals (that resembled the kidney study data intervals) around the survival times taken from the 1000 posterior predictive datasets. Next, using these intervals, we plotted the predicted Weibull cumulative density functions from the 1000 posterior datasets and then overlaid the estimated cumulative density curve from the raw data. We chose to plot the cumulative density function instead of the survival function because of the low event rate in these sub-groups [35]. We had to choose certain values of baseline covariates for these checks, so we presented three different types of individuals that represent the most prevalent combinations of covariates. The results of the survival predictive checks for three particular combinations of baseline covariates, for all three models M1-M3, are found in the Additional file 1: Figure S3. The coverage of the observed survival curve by the posterior predictive survival curves is best for M1. This is indicative that M1 fits the survival data better than the other two joint models.

### Sensitivity Analysis

To determine if the choice of priors affected the estimates of M1 parameters, an alternative prior distribution of Uniform (*U*(−100,100)) was compared against a Normal (*N*(0,1000)) prior for *b*_0_, *b*_1_, *β*, *λ*_0_ and *λ*_1_, and the half-Cauchy (*HC*(0,25)) distribution for *σ*_*e*_ was compared against the uniform prior (*U*(0,100)) for this variance component. The results, tabulated in Additional file 1: Table S1, indicate the choice of priors has very little effect on the parameter estimates.

### Simulation Study

We conducted a simulation study to compare how the joint M1 and the time varying covariate model perform under (1) varying degrees of association, (2) varying amounts of interval censoring (IC), and (3) varying amounts of measurement error (ME). We simulated data using the joint model with shared random intercepts (M2) under nine scenarios, each with a different combination of measurement error (none $\left [\sigma _{e}^{2}=0\right ]$, low $\left [\sigma _{e}^{2}=1\right ]$, and high $\left [\sigma _{e}^{2}=8\right ]$) and degree of interval censoring (lighter [0-1 month, 1–6 month, 6–12 month, 12–24 month, yearly after], visits missed at random, and heavier [3 year intervals]). For the random interval censoring, we assumed patients had a fixed follow-up schedule matching the lighter IC scenario, but missed 50% of visits at random (we also set the missing-ness to 25% and the results were almost identical). All other variables were set to be similar to the kidney transplant data, with Weibull parameters *α*=0.50 and *β*_0_=−2. Two hundred datasets were simulated under each of these six conditions, and the JM and TVC models were fitted to each dataset using MCMC. Each dataset contained 300 individuals, and approximately half developed dnDSA. The results from all simulations were obtained using two parallel chains; the TVC model had a burn-in of 1000 samples and 1000 samples for inference, while the JM had a burn-in of 2000 samples and 2000 samples for inference. The average model estimates from the light and heavy IC scenarios are reported in Table 4. The light and random IC scenarios did not differ significantly, so the results from the random IC scenarios are in the Additional file 1: Table S2.

**Table 4:** Results from the simulation study

		No Measurement Error			Low Measurement Error			High Measurement Error		
	Variable	True Value	M2	M4	True Value	M2	M4	True Value	M2	M4
Lighter interval censoring	*b*_1_	-0.03	-0.02 (0.00)		-0.03	-0.03 (0.005)		-0.03	-0.03 (0.005)	
	*b*_0_	7.00	6.92 (0.001)		7.00	7.02 (0.05)		7.00	6.99 (0.07)	
	$\sigma ^{2}_{e}$	0	0.00 (0.00)		1.00	1.00 (0.01)		8.00	8.01 (0.09)	
	*ρ*	-0.005	-0.01 (0.001)		-0.005	-0.005 (0.06)		-0.005	-0.001 (0.08)	
	$\sigma ^{2}_{0}$	1.75	1.76 (0.003)		1.75	1.76 (0.15)		1.75	1.75 (0.16)	
	$\sigma ^{2}_{1}$	0.004	0.004 (0.00)		0.004	0.004 (0.000)		0.004	0.004 (0.001)	
	*β*_0_, *γ*_0_	-2.00	-2.10 (0.006)	0.63 (0.009)	-2.00	-2.14 (0.26)	0.21 (0.39)	-2.00	-2.14 (0.27)	-0.82 (0.31)
	*β*_1_ (HLA)	0.25	0.26 (0.002)	0.24 (0.002)	0.25	0.26 (0.07)	0.24 (0.07)	0.25	0.26 (0.07)	0.23 (0.07)
	*α*	0.50	0.54 (0.00)	0.41 (0.00)	0.50	0.53 (0.04)	0.47 (0.04)	0.50	0.54 (0.04)	0.47 (0.04)
	*λ*_0_, *η*	-0.50	-0.52 (0.002)	-0.33 (0.001)	-0.50	-0.53 (0.07)	-0.32 (0.05)	-0.50	-0.54 (0.08)	-0.18 (0.04)
Heavier interval censoring	*b*_1_	-0.03	-0.02 (0.00)		-0.03	-0.03 (0.004)		-0.03	-0.03 (0.005)	
	*b*_0_	7.00	6.93 (0.00)		7.00	7.00 (0.05)		7.00	7.00 (0.08)	
	$\sigma ^{2}_{e}$	0	0.00 (0.00)		1.00	1.00 (0.01)		8.00	8.01 (0.09)	
	*ρ*	-0.005	-0.01 (0.001)		-0.005	-0.005 (0.06)		-0.005	-0.001 (0.08)	
	$\sigma ^{2}_{0}$	1.75	1.76 (0.003)		1.75	1.76 (0.15)		1.75	1.75 (0.16)	
	$\sigma ^{2}_{1}$	0.004	0.004 (0.00)		0.004	0.004 (0.000)		0.004	0.004 (0.001)	
	*β*_0_, *γ*_0_	-2.00	-3.99 (0.01)	-0.80 (0.01)	-2.00	-4.00 (0.49)	-1.61 (0.56)	-2.00	-4.20 (0.55)	-2.24 (0.49)
	*β*_1_ (HLA)	0.25	0.28 (0.002)	0.24 (0.002)	0.25	0.27 (0.09)	0.23 (0.08)	0.25	0.28 (0.09)	0.23 (0.08)
	*α*	0.50	1.08 (0.002)	0.69 (0.002)	0.50	1.09 (0.11)	0.87 (0.10)	0.50	1.15 (0.13)	0.89 (0.09)
	*λ*_0_, *η*	-0.50	-0.56 (0.002)	-0.26 (0.001)	-0.50	-0.59 (0.10)	-0.25 (0.06)	-0.50	-0.64 (0.12)	-0.18 (0.05)

Data were simulated from the joint model with shared random intercepts, M2, and used to fit M2 and M4 by MCMC. Six simulations were performed with varying amounts of measurement error (none ($\sigma _{e}^{2}=0$), low ($\sigma _{e}^{2}=1$), and high ($\sigma _{e}^{2}=8$)) and varying amounts of interval censoring (lighter (as in our data) and heavier (3 year intervals)). Unlike in Table 3, none of these estimates are converted into hazard ratios, because the interest here is comparing the results from the simulation to the true values. The numbers presented are mean (standard deviation) of the estimates from the 200 datasets for each simulation condition

**Fig. 2:**
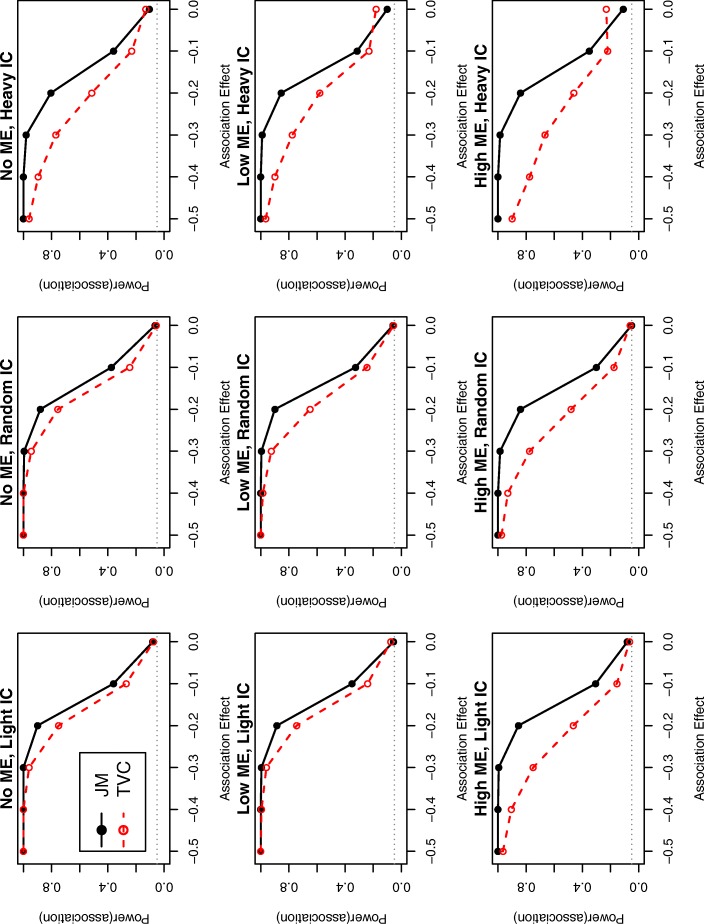
Power curves for each simulation scenario presented in Table 4. ME: measurement error. IC: interval censoring. Dotted grey line represents 0.05. Random IC: patients had the same follow-up schedule as the light IC, but randomly missed 50% of follow-up visits

To compare power for detecting an association between the longitudinal and the time to event outcomes, we varied the association between TAC and dnDSA (*λ*_0_ in the JM and *η* in the TVC) between -0.50 and 0 within each scenario. We calculated power based on each model at each true association value using the credible intervals of *λ*_0_ or *η*. For example, for simulation run *m*, *m*=1,...,*M* (M=200 for our simulations), let $\hat {\lambda }_{0L}$ and $\hat {\lambda }_{0U}$ denote the 2.5% and 97.5% percentiles of the distribution of the MCMC samples of $\hat {\lambda }_{0}$. The power is calculated as: Power $= 1-1/M\sum \limits _{m=1}^{M} I(\hat {\lambda }_{0L}<0<\hat {\lambda }_{0U})$, where I(E) is the indicator function of event E. The power curves for all nine scenarios are presented in Fig. 2.

As expected, the association parameter was consistently attenuated toward zero in the TVC compared to the JM. As more measurement error was introduced, the TVC underestimated the association even more (true value: *λ*_0_=−0.50, low ME: $\hat {\eta }$= -0.32 and high ME: $\hat {\eta }$= -0.18), while there was no evidence of substantial bias for the JM (low ME: $\hat {\lambda }_{0}= -0.53$ and high ME: $\hat {\lambda }_{0}= -0.54$). As heavier interval censoring was introduced, the association estimates were further from the true value in both modeling approaches, but the standard deviations also increased. The parameter that was most affected by heavier interval censoring was the Weibull shape parameter, *α*. Although the bias of *α* was lower in the TVC context, this model should not be preferred when there is high measurement error and heavy censoring, due to the underestimation of the association between the two outcomes, which is typically of more interest.

The power of the TVC model was consistently lower than the power of the JM (Fig. 2). When more measurement error was introduced, the power of the TVC model significantly decreased while the power of the JM stayed approximately the same. When visits were missed at random, the decrease in power on both approaches was minimal. The wider interval censoring around dnDSA resulted in a high type I error in the case of the TVC model (type I error=0.23 in the high ME and heavy IC simulation from Fig. 2). The average credible intervals and coverage probabilities for all simulations are reported in the Additional file 1: Figure S3.

## Discussion

In this paper, we compare two modeling techniques for two outcomes: a longitudinal outcome with high measurement error and a survival outcome with heavy interval censoring. For our motivating dataset, both DIC and WAIC suggested that a shared random effects model with random effects for both the intercept and slope of the longitudinal process (TAC) was the preferred model. Results from this model and from the TVC model (M4) support the hypothesis that the association between TAC and dnDSA is underestimated when a TVC model is fit. The simulation study further confirmed this hypothesis and showed that the TVC has lower power and higher type I error compared to the JM when the outcomes have high measurement error and heavy interval censoring.

TAC is the most commonly used immunosuppressant drug in kidney transplantation and yet optimal therapeutic dose to prevent dnDSA, a common reason for graft loss, is unknown. Most published studies on the influence of immunosuppressant drugs on development of dnDSA compare drugs rather than evaluate dosing of a specific drug [36, 37]. A study in the liver transplant setting by Kaneku et al. analyzed risk factors for dnDSA in 749 adult liver transplant recipients [38]. They found 8.1% of individuals developed dnDSA at one year and at least one low TAC (<3 ng/ml) or cyclosporine (<75 ng/ml) level were associated with dnDSA (OR 2.66, p = 0.015). In our dataset, 21.7*%* of individuals had dnDSA by 1 year and we also found that for every one ng/ml lower average TAC the risk of dnDSA was higher (HR [95% CrI]: 1.56 [1.33, 1.92]) and the hazard of dnDSA also increased 2.33-fold (95% CrI: 1.72, 3.13) for every 0.05 ng/ml/month decrease in the slope of TAC. The higher levels of dnDSA detected in our study may be due to several reasons, including center differences in detection levels of antibody counted as positive and inclusion criteria. Advancing previous models, we modeled the time to dnDSA while accounting for changes in TAC, where TAC is modeled as a variable with measurement error. Although minimization of TAC exposure with lower drug trough levels may decrease its associated toxicities, our results suggest that this strategy may place individuals at increased risk for dnDSA, which may have important implications for intermediate and long-term graft survival.

Similar to the findings presented by Kolagmunnage and Prentice [8, 9], our simulation found severe underestimation of the association between the longitudinal and survival outcomes when using the time-varying covariate model for longitudinal data with a considerable amount of measurement error. We also found a similarly severe underestimation of the association using the TVC model when the survival outcome was interval censored, even in the case of no measurement error. Importantly, the TVC model had a lower power to detect an association compared to the JM, and it had a higher type I error rate when heavy interval censoring was introduced. Interestingly, when half of the follow-up visits were missed at random for the scenarios studied, there was a negligible overall effect on power for both the TVC and JM. These results are important in understanding the behavior of the JM and TVC since in many clinical settings time to event outcomes can often be observed only within intervals. In future work, we would like to study the effect of informative missing-ness (e.g. probability of missing depends on a covariate, such as age, or the probability of missing increases as time from transplant progresses) on the power of detecting associations within these models.

We recognize the inherent differences in the two modeling approaches discussed here. The joint models (M1-M3) all result in between-subject association interpretations. For example, *λ*_0_ is interpreted as the increased hazard of dnDSA for an individual with an average TAC level of one ng/ml lower than another individual. In contrast, the time-varying covariate model (M4) yields a within-subject interpretation. In this model, *η* relates to the increased hazard of dnDSA for a given individual, immediately following a one ng/ml decrease in TAC.

By employing MCMC to fit all models, we were able to easily obtain the posterior distribution of all estimated parameters. Since the main focus of this analysis was to assess the association between TAC and dnDSA, we were particularly interested in the association parameters: *λ*_0_ and *λ*_1_ in M1 and *η* in M4. We calculated credible intervals (CrI) for these parameters, which give the straightforward interpretation as an interval that contains the association parameter with 95% certainty. The MCMC framework also allows for easy calculations of dynamic predictions and this will be explored in future work. The code to fit the final joint model (M1) and time-varying covariate model (M4) on simulated data in JAGS is located in the Additional file 2. The code for fitting M1 in SAS using PROC NLMIXED is also provided.

## Conclusion

The joint model examined in this paper allows for flexible modeling of dnDSA development as it relates to TAC levels over time. As our simulation study showed, this approach accommodates modeling heavily interval censored survival data jointly with highly variable longitudinal data, and if a time varying covariate (TVC) approach is used with heavy interval censoring and high measurement error, the associations may be severely underestimated, missed completely, or detected spuriously.

## Additional files

Additional file 1 Supplemental materials. Contains details on likelihood construction, additional figures and tables, and selected output from the reproducible examples. (PDF 393 kb)

Additional file 2 ∙ example-1-JM.R: Code to fit M1
∙ longitudinal-data.csv: simulated TAC data for M1
∙ survival-data.csv: simulated dnDSA data for M1
∙ model-1-JM.txt: JAGS model, called by example-1-JM.R
∙ example-1-NLMIXED.SAS: Code to fit M1 with PROC NLMIXED, uses same simulated data
∙ example-4-TVC.R: Code to fit M4
∙ longitudinal data tvc.csv: simulated TAC data for M4 (carried forward values of TAC)
∙ model-4-TVC.txt: JAGS model, called by example-4-TVC.R (ZIP 199 kb)

## Data Availability

Simulated datasets and the code used to fit the joint model and time varying covariate model are publicly available in the Additional file 2.

## Abbreviations

CrI: Credible interval
DIC: Deviance information criterion
dnDSA: de novo Donor specific antibody
HLA: Human leucocyte antigen
IC: Interval censoring
JAGS: Just another Gibbs sampler
JM: Joint model
MCMC: Markov chain Monte Carlo
ME: Measurement error
TAC: Tacrolimus
TVC: Time-varying covariate
WAIC: Watanabe-Akaike information criterion
