# Supplementary material for: Comparison of a time-varying covariate model and a joint model of time-to-event outcomes in the presence of measurement error and interval censoring: application to kidney transplantation
Source: BMC Med Res Methodol. 2019 Jun 26;19:130. doi: 10.1186/s12874-019-0773-1 (PMC6595621; doi:10.1186/s12874-019-0773-1)
Supplement: Supplementary file 1 — Supplemental materials. Contains details on likelihood construction, additional figures and tables, and selected output from the reproducible examples. (PDF 393 kb) [file 12874_2019_773_MOESM1_ESM.pdf]

Joint Modeling of Longitudinal and Interval Censored Time-to-Event Outcomes: Application to Tacrolimus and Antibody Formation in Kidney Transplant Patients
Additional File 1

K. Campbell, E. Juarez-Colunga, GK. Grunwald, J. Cooper, S. Davis, and J. Gralla

June 17, 2019

Weibull hazard function:

$$h_{it} = \alpha \exp(\beta_0 + \beta_2' x_{2i} + \lambda_0 a_{0i} + \lambda_1 a_{1i}) t^{\alpha-1} \quad (1)$$

Likelihood Construction:

The log likelihood for the ith subject is written as:

$$LL_i = \sum_{j=1}^{n_i} \{-\frac{1}{2} ln(2\pi\sigma_e^2) - \frac{1}{2\sigma_e^2}(y_{ij} - b_0 - b_1 t_{ij} - \beta_1' x_{1i} - a_{0i} - a_{1i} t_{ij})^2\}$$

$$-I_{Ri} ln\{1 - F_i(t_{Ri})\} + (1 - I_{Ri}) ln\{F_i(t_{Ri}) - F_i(t_{Li})\}$$

Note that $H(t) = -ln[1 - F(t)]$, so the contribution for right censored patients is:

$$I_{Ri} ln\{1 - F_i(t_{Ri})\} = -I_{Ri}[\exp\{\beta_0 + \beta_2' x_{2i} + \lambda_0 a_{0i} + \lambda_1 a_{1i}\} t_{Ri}^{\alpha}]$$

Also, note that $F(t) = 1 - \exp\{-H(t)\}$, so the contribution for interval censored patients is:

$$I_{I_i} ln\{F_i(t_{Ri}) - F_i(t_{Li})\} = \delta_{I_i} ln\{e^{-H(t_{Li})} - e^{-H(t_{Ri})}\}$$

$$= I_{I_i} ln[\exp\{\exp\{\beta_0 + \beta_2' x_{2i} + \lambda_0 a_{0i} + \lambda_1 a_{1i}\} t_{Li}^{\alpha}\} - \exp\{-\exp\{\beta_0 + \beta_2' x_{2i} + \lambda_0 a_{0i} + \lambda_1 a_{1i}\} t_{Ri}^{\alpha}\}]$$

So, all together:

$$LL_i = \sum_{j=1}^{n_i} \{-\frac{1}{2} ln(2\pi\sigma_e^2) - \frac{1}{2\sigma_e^2}(y_{ij} - b_0 - b_1 t_{ij} - \beta_1' x_{1i} - a_{0i} - a_{1i} t_{ij})^2\}$$

$$-I_{Ri}[\exp\{\beta_0 + \beta_2' x_{2i} + \lambda_0 a_{0i} + \lambda_1 a_{1i}\} t_{Ri}^{\alpha}]$$

$$= I_{I_i} ln[\exp\{-\exp\{\beta_0 + \beta_2' x_{2i} + \lambda_0 a_{0i} + \lambda_1 a_{1i}\} t_{Li}^{\alpha}\} - \exp\{-\exp\{\beta_0 + \beta_2' x_{2i} + \lambda_0 a_{0i} + \lambda_1 a_{1i}\} t_{Ri}^{\alpha}\}]$$

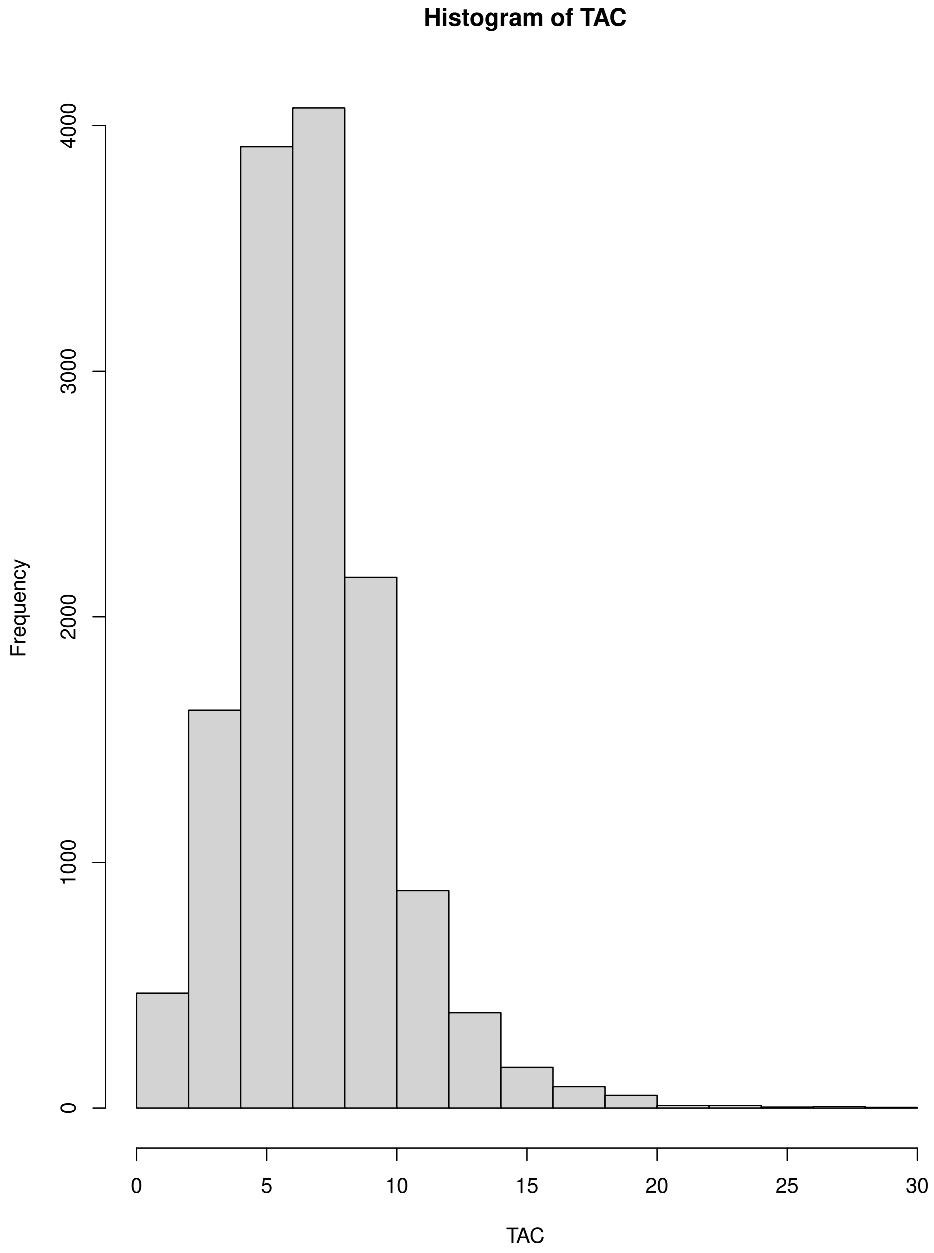

Figure S1: Distribution of TAC

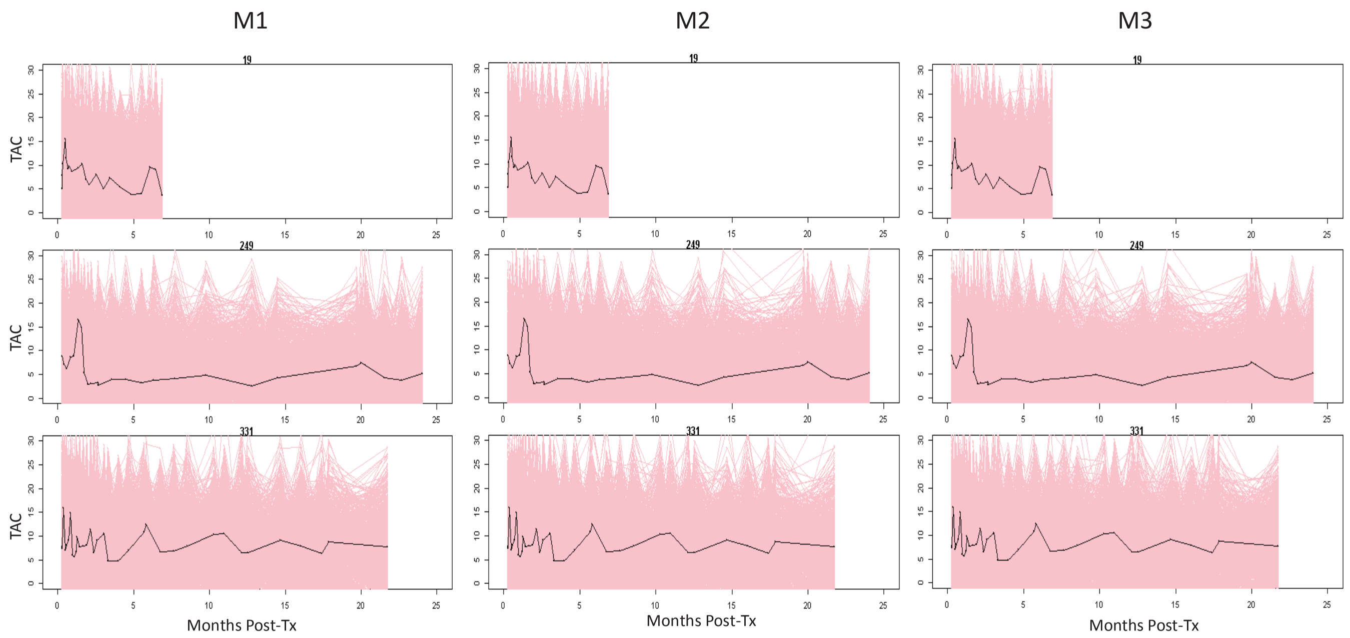

Figure S2: Posterior Predictive Trajectories and the observed trajectory for 3 individuals for the three joint models, M1-M3

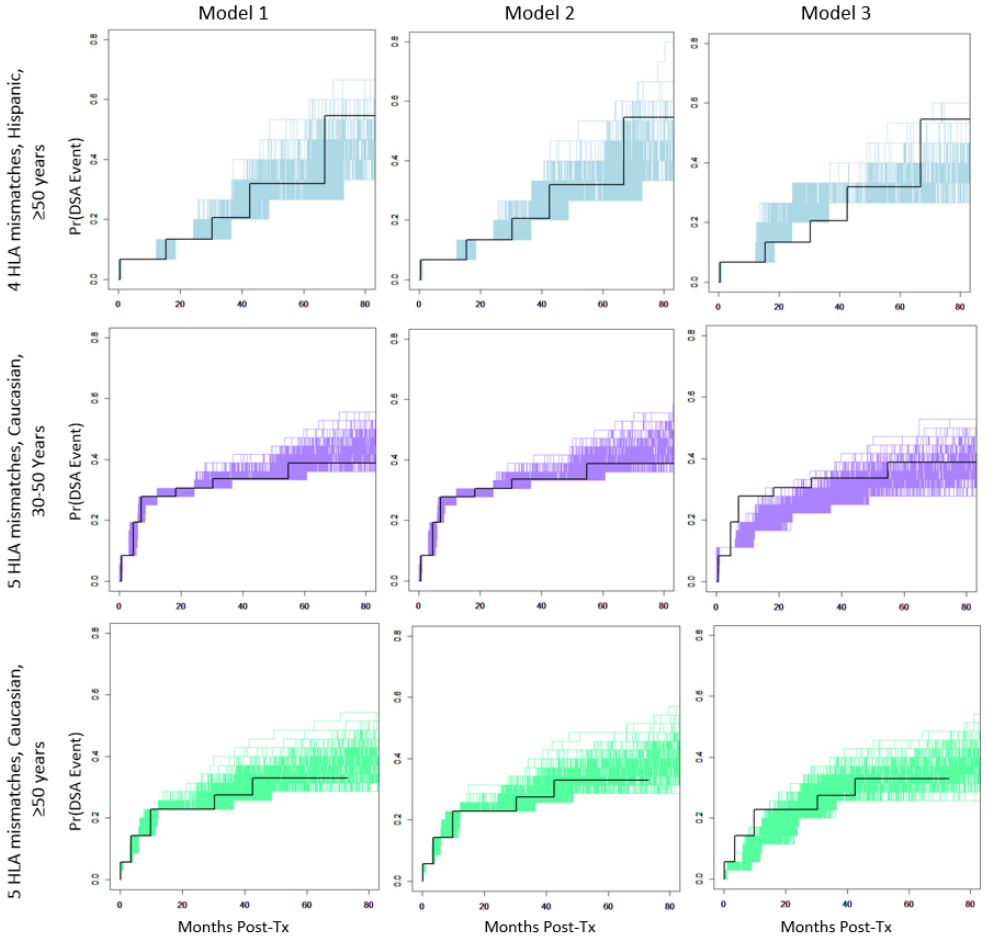

Figure S3: Posterior predictive interval censored cumulative density curve and the observed interval censored cumulative density curve for 3 types of subjects for the 3 joint models M1, M2, and M3.

Table S1: Results from sensitivity analysis. First column is the results from the actual model that was fit in the Results section of the paper. This has normal priors for regression and association parameters and a uniform distribution for the standard deviation of the random error term.

	Model 1	Uniform Prior Regression/Loading Params	Half-Cauchy Prior for σ_ϵ^2
	Mean (95% CrI)	*Mean (95% CrI)*	*Mean (95% CrI)*
Linear			
b_0	7.24 (7.12, 7.37)	7.24 (7.11, 7.36)	7.24 (7.12, 7.37)
b_1	-0.05 (-0.06, -0.04)	-0.05 (-0.06, -0.04)	-0.05 (-0.06, -0.04)
σ_ϵ^2	7.44 (7.26, 7.63)	7.37 (7.19, 7.56)	7.37 (7.19, 7.55)
ρ	-0.38 (-0.52, -0.24)	-0.40 (-0.52,-0.26)	-0.40 (-0.52, -0.26)
σ_0^2	1.66 (1.40, 1.94)	1.73 (1.47, 2.01)	1.73 (1.47, 2.04)
σ_1^2	0.004 (0.003, 0.005)	0.004 (0.003, 0.005)	0.004 (0.003, 0.005)
Survival			
α	0.57 (0.47, 0.69)	0.55 (0.46, 0.67)	0.55 (0.46, 0.65)
	HR (95% CrI)	*HR (95% CrI)*	*HR (95% CrI)*
β_0	0.03 (0.01, 0.06)	0.03 (0.02, 0.06)	0.03 (0.02, 0.06)
β_1 (HLA Mismatch Number)	1.26 (1.13, 1.40)	1.28 (1.15, 1.42)	1.28 (1.15, 1.42)
β_2 (African American)	2.09 (1.23, 3.39)	2.02 (1.17, 3.25)	2.00 (1.16, 3.13)
β_3 (Hispanic)	1.60 (1.05, 2.31)	1.64 (1.04, 2.40)	1.64 (1.07, 2.39)
β_4 (Other Race)	1.14 (0.34, 2.41)	1.23 (0.41, 2.61)	1.24 (0.41, 2.67)
β_5 (30-49 years)	0.57 (0.32, 0.93)	0.60 (0.34, 1.00)	0.60 (0.34, 0.99)
β_6 ($\geq$50 years)	0.30 (0.17, 0.49)	0.32 (0.18, 0.54)	0.32 (0.18, 0.54)
λ_0	0.64 (0.52, 0.75)	0.65 (0.53, 0.77)	0.65 (0.54, 0.77)
λ_1	0.43 (0.32, 0.58)	0.47 (0.32, 0.63)	0.48 (0.33, 0.63)

Table S2: Results from the simulation study - random interval censoring. Data were simulated from the joint model with shared random intercepts, M2, and used to fit M2 and M4 by MCMC. Three simulations were performed with varying amounts of measurement error (none ($\sigma_e^2 = 0$), low ($\sigma_e^2 = 1$), and high ($\sigma_e^2 = 8$)). The follow-up visit process was set at 1 month, 6 month, 12 month, 24 months, and yearly after. Patients missed any arbitrary visit with 50% probability. Unlike in Table **??**, none of these estimates are converted into hazard ratios, because the interest here is comparing the results from the simulation to the true values. The numbers presented are mean (standard deviation) of the estimates from the 200 datasets for each simulation condition.

		No Measurement Error			Low Measurement Error			High Measurement Error		
	Variable	True Value	M2	M4	True Value	M2	M4	True Value	M2	M4
Random interval censoring	b_1	-0.03	-0.02 (0.00)		-0.03	-0.03 (0.00)		-0.03	-0.03 (0.0001)	
	b_0	7.00	6.93 (0.001)		7.00	6.98 (0.001)		7.00	7.02 (0.001)	
	σ_e^2	0	0.00 (0.00)		1.00	1.00 (0.0001)		8.00	8.00 (0.002)	
	ρ	-0.005	-0.01 (0.001)		-0.005	-0.004 (0.001)		-0.005	-0.0004 (0.002)	
	σ_0^2	1.75	1.76 (0.003)		1.75	1.75 (0.003)		1.75	1.75 (0.004)	
	σ_1^2	0.004	0.004 (0.00)		0.004	0.004 (0.000)		0.004	0.004 (0.000)	
	β_0, γ_0	-2.00	-2.29 (0.007)	0.64 (0.01)	-2.00	-2.30 (0.007)	0.45 (0.01)	-2.00	-2.34 (0.007)	-0.47 (0.008)
	β_1 (HLA)	0.25	0.27 (0.002)	0.24 (0.002)	0.25	0.26 (0.002)	0.24 (0.002)	0.25	0.27 (0.002)	0.23 (0.002)
	α	0.50	0.59 (0.001)	0.40 (0.00)	0.50	0.58 (0.001)	0.41 (0.00)	0.50	0.59 (0.001)	0.42 (0.00)
	λ_0, η	-0.50	-0.52 (0.002)	-0.33 (0.001)	-0.50	-0.53 (0.002)	-0.31 (0.001)	-0.50	-0.54 (0.002)	-0.19 (0.00)

Table S3: Additional Simulation Study Results: Credible Intervals and Coverage Probabilities. The true value of the association parameter was set to -0.50. The average credible interval (CrI) from 200 simulations is reported for each model (M2 and M4) for each simulation scenario presented in Table 4. The coverage probability was calculated as the percentage of simulations that had the true value, -0.50, within the calculated credible interval. ME: measurement error. IC: interval censoring.

Simulation Scenario	Model	Average Credible Interval	Coverage Probability
(1) No ME, Low IC	JM (M2)	(-0.659, -0.381)	93%
	TVC (M4)	(-0.432, -0.220)	26%
(2) Low ME, Low IC	JM (M2)	(-0.661, -0.382)	94%
	TVC (M4)	(-0.402, -0.199)	15%
(3) High ME, Low IC	JM (M2)	(-0.688, -0.376)	92%
	TVC (M4)	(-0.240, -0.087)	0%
(4) No ME, Random IC	JM (M2)	(-0.679, -0.376)	93%
	TVC (M4)	(-0.446, -0.217)	30%
(5) Low ME, Random IC	JM (M2)	(-0.684, -0.377)	95%
	TVC (M4)	(-0.423, -0.201)	24%
(6) High ME, Random IC	JM (M2)	(-0.710, -0.372)	93%
	TVC (M4)	(-0.273, -0.103)	0%
(7) No ME, High IC	JM (M2)	(-0.745, -0.378)	88%
	TVC (M4)	(-0.379, -0.145)	15%
(8) Low ME, High IC	JM (M2)	(-0.775, -0.395)	86%
	TVC (M4)	(-0.385, -0.151)	20%
(9) High ME, High IC	JM (M2)	(-0.857, -0.408)	81%
	TVC (M4)	(-0.293, -0.100)	2%

Model 1 (JM) Reproducible Example:

```
## selecting only the parameters of interest to monitor (excluding likelihood)
vars<-mcmc.list(res_jm[[1]][,c(1:17)],res_jm[[2]][,c(1:17)])
summary(vars)

1. Empirical mean and standard deviation for each variable,
plus standard error of the mean:

                     Mean        SD  Naive SE Time-series SE
b0               6.954070 7.452e-02 8.332e-04      5.949e-03
b1              -0.027505 4.086e-03 4.568e-05      7.215e-04
sige             1.000249 5.776e-03 6.458e-05      9.269e-05
precision        0.999602 1.154e-02 1.291e-04      1.851e-04
prec_inv         1.000532 1.156e-02 1.292e-04      1.855e-04
rho.0r           0.067617 6.175e-02 6.904e-04      1.076e-03
sig.bc[1]        1.654051 1.412e-01 1.578e-03      2.346e-03
sig.bc[2]        0.003584 3.314e-04 3.705e-06      7.179e-06
omega.bc[1,1]    0.614141 5.224e-02 5.841e-04      9.199e-04
omega.bc[2,1]   -0.896581 8.230e-01 9.202e-03      1.456e-02
omega.bc[1,2]   -0.896581 8.230e-01 9.202e-03      1.456e-02
omega.bc[2,2]  283.773615 2.606e+01 2.914e-01      6.032e-01
bet0            -2.043759 2.746e-01 3.070e-03      2.326e-02
bet_hla          0.150093 7.159e-02 8.004e-04      5.655e-03
alpha            0.582307 4.751e-02 5.312e-04      2.914e-03
lam0            -0.592618 7.137e-02 7.980e-04      2.312e-03
lam1             0.428651 1.557e+00 1.741e-02      4.683e-02

2. Quantiles for each variable:

                     2.5%        25%        50%        75%      97.5%
b0               6.841771   6.885393   6.963856   7.022935   7.069696
b1              -0.035899  -0.030211  -0.027352  -0.024809  -0.019634
sige             0.989011   0.996391   1.000232   1.004136   1.011744
precision        0.976920   0.991780   0.999536   1.007256   1.022346
prec_inv         0.978142   0.992796   1.000464   1.008289   1.023625
rho.0r          -0.053057   0.025121   0.067684   0.109759   0.185387
sig.bc[1]        1.397020   1.557228   1.647406   1.742156   1.957136
sig.bc[2]        0.002987   0.003351   0.003566   0.003793   0.004286
omega.bc[1,1]    0.515482   0.578846   0.612195   0.647450   0.721196
omega.bc[2,1]   -2.496444  -1.444929  -0.890590  -0.330632   0.695669
omega.bc[1,2]   -2.496444  -1.444929  -0.890590  -0.330632   0.695669
omega.bc[2,2]  235.077989 265.709530 282.707070 300.972316 337.013906
bet0            -2.591134  -2.223170  -2.044304  -1.861695  -1.499589
bet_hla          0.006230   0.102881   0.150280   0.197456   0.286429
```

```
alpha            0.490768   0.549416   0.581517   0.615312   0.677390
lam0            -0.738735  -0.638706  -0.592066  -0.545223  -0.456393
lam1            -2.645301  -0.615691   0.404060   1.478742   3.483264

## plots
traplot(vars)
autocorr.plot(vars)

## tests of convergence
geweke.diag(vars)

[[1]]

Fraction in 1st window = 0.1
Fraction in 2nd window = 0.5

b0              b1              sige            precision       prec_inv
2.13707        -0.37271        -0.91748         0.92775        -0.91411

omega.bc[1,1]   omega.bc[2,1]   omega.bc[1,2]   omega.bc[2,2]
1.72798          1.36731         1.36731        -0.50256

rho.0r          sig.bc[1]       sig.bc[2]
-1.41746        -1.66230        0.43216

bet0            bet_hla         alpha           lam0            lam1
-1.48643        1.15813         0.28768         1.01478         0.02158


[[2]]

Fraction in 1st window = 0.1
Fraction in 2nd window = 0.5

b0              b1              sige            precision       prec_inv
-2.11622        -2.07081         1.42478       -1.43566         1.42101

omega.bc[1,1]   omega.bc[2,1]   omega.bc[1,2]   omega.bc[2,2]
-2.84444         2.73654         2.73654        -0.83759

rho.0r          sig.bc[1]       sig.bc[2]
-2.52219        2.79384         0.72391

bet0            bet_hla         alpha           lam0            lam1
-0.07517        -0.13393        0.79316         1.20012         -1.73095
```

```
## calculate WAIC
waic.jm1.long <- waicf(res_jm,18,317)
waic.jm1.surv <- waicf(res_jm,318,617)
> print(waic.jm1.long)
$waic
[1] 44129.07

$p_waic
[1] 291.8147

$lppd
[1] -21772.72

$p_waic_1
[1] 180.8082

> print(waic.jm1.surv)
$waic
[1] 578.6873

$p_waic
[1] 6.348036

$lppd
[1] -282.9956

$p_waic_1
[1] 6.056737
```

Model 4 (TVC) Reproducible Example:

```
## selecting only the parameters of interest to monitor (excluding likelihood)
vars<-mcmc.list(res_tvc[[1]][,c(1:4)],res_tvc[[2]][,c(1:4)])
summary(vars)


Iterations = 2001:3000
Thinning interval = 1
Number of chains = 2
Sample size per chain = 1000

1. Empirical mean and standard deviation for each variable,
plus standard error of the mean:

             Mean      SD  Naive SE Time-series SE
gam0      -0.8024 0.36720 0.025965       0.146913
eta       -0.1505 0.04332 0.003063       0.011836
alpha_tvc  0.2655 0.02333 0.001649       0.002712
gam_hla    0.1216 0.05833 0.004124       0.015049

2. Quantiles for each variable:

              2.5%      25%     50%     75%    97.5%
gam0      -1.545118 -1.05912 -0.7470 -0.5376 -0.24187
eta       -0.217281 -0.18440 -0.1565 -0.1128 -0.07603
alpha_tvc  0.218658  0.25255  0.2631  0.2796  0.31636
gam_hla    0.008646  0.07885  0.1232  0.1648  0.21983

## plots
traplot(vars)
autocorr.plot(vars)

## tests of convergence
gelman.diag(vars)

Potential scale reduction factors:

          Point est. Upper C.I.
gam0            1.45       2.72
eta             1.83       3.62
alpha_tvc       1.01       1.04
gam_hla         1.07       1.09

Multivariate psrf
```

```
1.44

geweke.diag(vars)

[[1]]

Fraction in 1st window = 0.1
Fraction in 2nd window = 0.5

gam0       eta       alpha_tvc   gam_hla
3.94994   -0.01095   -0.24655   -6.33207


[[2]]

Fraction in 1st window = 0.1
Fraction in 2nd window = 0.5

gam0       eta        alpha_tvc   gam_hla
-1.1836    2.6042     1.8018      -0.2949

##### calculating WAIC
waic.tvc <- waicf(res_tvc,5,304)
print(waic.tvc)

$waic
[1] 805.4979

$p_waic
[1] 3.490612

$lppd
[1] -399.2584

$p_waic_1
[1] 3.394789
```

NOTE: GCONV convergence criterion satisfied.

Fit Statistics	
-2 Log Likelihood	35778
AIC (smaller is better)	35800
AICC (smaller is better)	35800
BIC (smaller is better)	35841

Parameter Estimates								
Parameter	Estimate	Standard Error	DF	t Value	Pr > |t|	95% Confidence Limits		Gradient
b0	6.9690	0.07415	298	93.99	<.0001	6.8231	7.1149	0.054314
b1	-0.02734	0.003704	298	-7.38	<.0001	-0.03463	-0.02006	-1.16318
sige	1.0001	0.005809	298	172.17	<.0001	0.9887	1.0115	-0.20501
bet0	-2.0226	0.2717	298	-7.44	<.0001	-2.5573	-1.4879	0.35361
bet_hla	0.1468	0.07199	298	2.04	0.0423	0.005171	0.2885	-0.26264
lam0	-0.5915	0.07033	298	-8.41	<.0001	-0.7299	-0.4531	-0.71404
lam1	0.4296	1.6082	298	0.27	0.7895	-2.7351	3.5944	0.014834
alpha	0.5811	0.04726	298	12.30	<.0001	0.4881	0.6741	-0.19330
s2a0	1.6181	0.1325	298	12.21	<.0001	1.3573	1.8790	-1.41568
s2a1	0.003561	0.000329	298	10.81	<.0001	0.002913	0.004209	7.42673
cov	0.005037	0.004719	298	1.07	0.2866	-0.00425	0.01432	-1.70062

Model 1 (JM) Using PROC NLMIXED
